# Rapid Detection of Aluminium and Iron Impurities in Lithium Carbonate Using Water-Soluble Fluorescent Probes

**DOI:** 10.3390/molecules30010135

**Published:** 2024-12-31

**Authors:** Hong-Mei Wu, Huai-Gang Cheng, Zi-Wen Zhu, Li Cui

**Affiliations:** 1 Salt Lake Chemical Engineering Research Complex, Qinghai University, Xining 810016, China; 13007085087@163.com; 2 Institute of Resources and Environmental Engineering, Shanxi University, Taiyuan 030032, China; zhuziwenhi@163.com (Z.-W.Z.); cuili.123@163.com (L.C.)

**Keywords:** lithium carbonate, fluorescent probes, water solubility, metal ions, detection

## Abstract

The real-time measurement of the content of impurities such as iron and aluminium ions is one of the keys to quality evaluation in the production process of high-purity lithium carbonate; however, impurity detection has been a time-consuming process for many years, which limits the optimisation of the production of high-purity lithium carbonate. In this context, this work explores the possibility of using water-soluble fluorescent probes for the rapid detection of impurity ions. Salicylaldehyde was modified with the hydrophilic group dl-alanine to synthesise a water-soluble Al^3+^ fluorescent probe (Probe A). Moreover, a water-soluble Fe^3+^ fluorescent probe (Probe B) was synthesised from coumarin-3-carboxylic acid and 3-hydroxyaminomethane. Probe A and Probe B exhibited good stability in the pH range of 4–9 in aqueous solutions, high sensitivity, as well as high selectivity for Al^3+^ and Fe^3+^; the detection limits for Al^3+^ and Fe^3+^ were 1.180 and 1.683 μmol/L, whereas the response times for Al^3+^ and Fe^3+^ were as low as 10 and 30 s, respectively. Electrostatic potential (ESP) analysis and density functional theory calculations identified the binding sites and fluorescence recognition mechanism; theoretical calculations showed that the enhanced fluorescence emission of Probe A when detecting Al^3+^ was due to the excited intramolecular proton transfer (ESIPT) effect, whereas the fluorescence quenching of Probe B when detecting Fe^3+^ was due to the electrons turning off fluorescence when binding through the photoelectron transfer (PET) mechanism.

## 1. Introduction

Lithium carbonate is widely used in many fields, owing to its unique electrochemical reactivity. The development of the new energy industry has created an increasing demand for high-purity lithium carbonate, the manufacture of which has become an active area of research [1]. However, impurities such as iron and aluminium in the produced lithium carbonate can affect its purity, electrochemical performance, and thermal stability [2,3]. The purification of lithium carbonate is typically carried out in an aqueous environment, but the real-time monitoring of impurities during this process has been challenging. This is partly because impurity ions in the aqueous environment are difficult to label and track in real-time using traditional tracer methods.

Crude lithium carbonate collected from salt lakes can be contaminated with impurities such as iron and aluminium, due to equipment corrosion by brine. During the production process, alloy ions such as iron and aluminium can enter the lithium salt, which can subsequently affect the use of high-purity lithium carbonate owing to the paramagnetic properties of iron, the poor coordination ability of aluminium, and its strong hydration tendency. Therefore, it is important to develop a method for the rapid detection of Al^3+^ and Fe^3+^ in high-purity lithium carbonate and to detect impurity metal ions such as Al^3+^ and Fe^3+^ in the process of lithium carbonate production. Current detection methods for Al^3+^ and Fe^3+^ in high-purity lithium carbonate include atomic absorption spectroscopy (AAS), inductively coupled plasma emission spectrometry (ICP-OES), and inductively coupled plasma mass spectrometry (ICP-MS). Conventional methods are mainly suitable for the routine detection of substance content and have slow detection speeds; the pretreatment work is tedious and cannot realize real-time detection on site. Furthermore, these methods cannot accurately describe the distribution and dynamic changes of the measured metal ions in the lithium carbonate matrix [4,5].

Compared with the above methods, the fluorescence technique is the most attractive and sensitive approach for detecting analytes at low concentrations [6]; the essence of the fluorescence probe detection method is the specific interaction between fluorescent substances and molecules or ions, which can emit fluorescence at specific wavelengths under ultraviolet or visible light irradiation [7,8]. The fluorescent probe method is more suitable for the analysis of trace metal ions in complex matrices, owing to its high sensitivity, good selectivity, and fast response [9,10,11,12,13]. In recent years, considerable progress has been made in the detection of Al^3+^ and Fe^3+^ using fluorescent chemical sensors [14,15,16]. However, most of the reported fluorescent probes still suffer from many limitations affecting their application, such as the probe being insoluble in water and unable to operate in an aqueous environment. Probe molecules that are slightly soluble in water have poor selectivity or sensitivity and weak binding abilities [17,18,19,20], which greatly limits the application of Al^3+^ and Fe^3+^ sensors in aqueous environments [21,22,23,24,25,26,27]. Therefore, the design and synthesis of a high-sensitivity and high-selectivity sensor for trace Al^3+^ and Fe^3+^ in water is a highly important goal. The key to the rapid detection of trace amounts of Al^3+^ and Fe^3+^ in high-purity lithium carbonate by fluorescent probe methods is to fabricate water-soluble fluorescent probes suitable for high-concentration salt systems.

The objectives of this work are to introduce hydrophilic groups to fabricate water-soluble probes, use the C, O, N, and other atoms in the fluorescent probe to provide binding sites for Al^3+^ and Fe^3+^, and detect metal ions in high-salt systems during the production process based on changes in fluorescence intensity.

The hydrophilic group dl-alanine was employed to modify salicylaldehyde and prepare a 100% water-soluble Al^3+^ fluorescent probe (Probe A), whereas coumarin-3-carboxylic acid and 3-hydroxyaminomethane were used to synthesise a 100% water-soluble Fe^3+^ fluorescent probe (Probe B), aiming to achieve a rapid quality evaluation of lithium carbonate in a water-soluble environment, simplify the detection steps, and accelerate the detection of the impurities Al^3+^ and Fe^3+^ in high-purity lithium carbonate.

## 2. Results and Discussion

### 2.1. Synthesis of Water-Soluble Probe A and Probe B

**Figure 1:**
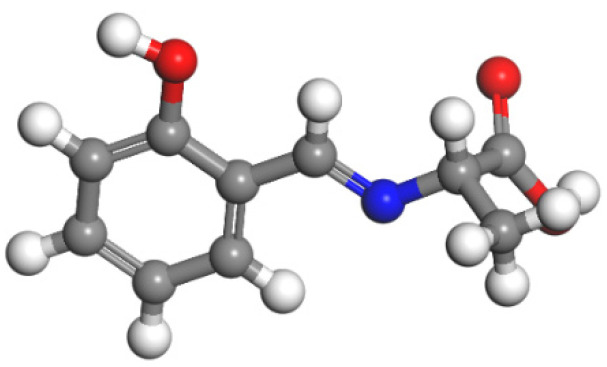
Structure of probe A. (In the figure, red, white, gray and blue correspond to oxygen, hydrogen, carbon and nitrogen, respectively.)

A Schiff base was synthesised by microwave irradiation [28,29]. First, DL-alanine (2.5 mmol) was dissolved in 20 mL of ethanol. After its complete dissolution, a 5A-grade molecular sieve was added; then, 10 mL of ethanol and 2 mmol of salicylaldehyde were slowly added dropwise with ultrasonic shaking for 1.5 h. After the reaction, filtration was carried out, and the filtrate was recrystallised with absolute ethanol to obtain a yellow-green powder. The yield was 83%. The structure is shown in Figure 1.

**Figure 2:**
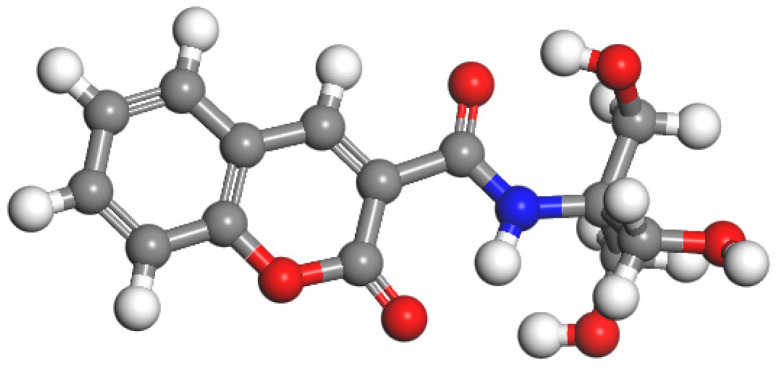
Structure of Probe B. (In the figure, red, white, gray and blue correspond to oxygen, hydrogen, carbon and nitrogen, respectively.)

Coumarin-3 carboxylic acid (190.15 mg, 1 mmol), 1-Ethyl-(3-dimethylaminopropyl) carbodiimide hydrochloride (233.7 mg, 1.2 mmol), 1-hydroxybenztriazole (1646.6 mg, 1.2 mmol), and dichloromethane (20 mL) were added to a reaction flask at room temperature and stirred under N_2_ protection for 1 h. After 1 h, 350 μL of triethylamine (2.5 mmol) and 121.8 mg (1 mmol) of 3-hydroxyaminomethane were added and stirred for 24 h. After the reaction was completed, a crude product was obtained, and a chromatography column (developing agent: ethyl acetate/methanol = 3:1) was used to obtain the target compound with a yield of 57%. The structure is shown in Figure 2.

### 2.2. Selective Identification of Probe A and Probe B

**Figure 3:**
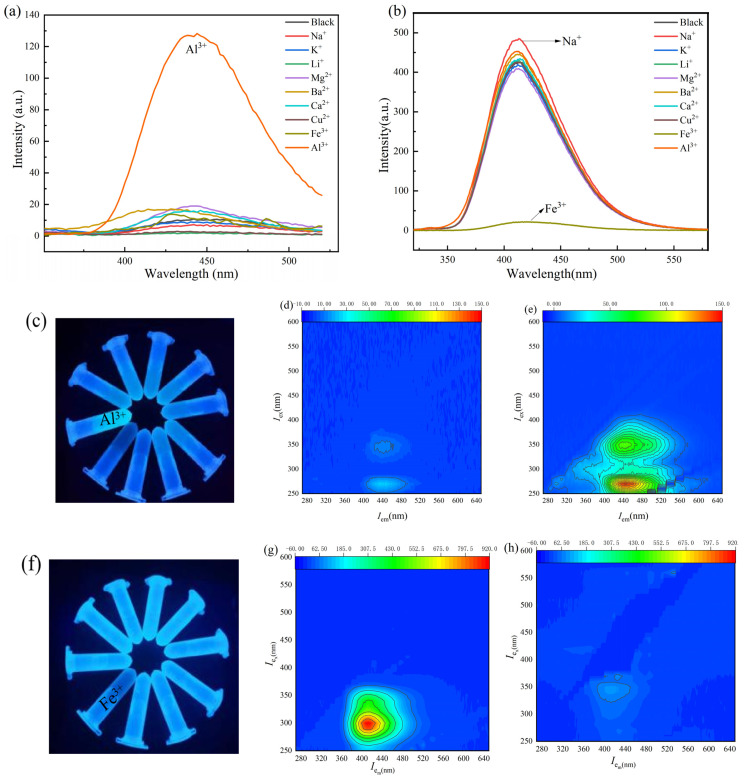
(**a**,**b**) Effect of different metal ions on the fluorescence spectrum of the probe; (**c**,**f**) photographs of probes after the addition of different metal ions under a 365 nm UV lamp; (**d**,**g**) three-dimensional fluorescence spectra of Probe A and Probe B aqueous solutions; (**e**,**h**) three-dimensional fluorescence spectra of Probe A/Al^3+^ = 1:1 and Probe B/Fe^3+^ = 1:1 aqueous solutions.

The ability of the probes to bind to metal ions was investigated by testing the selectivity of Probe A and Probe B for different metal ions in H_2_O solution. The results are shown in Figure 3.

As shown in Figure 3a, 3 mL of solutions of different metal cations (Na^+^, K^+^, Li^+^, Mg^2+^, Ba^2+^, Ca^2+^, Cu^2+^, Fe^3+^, Al^3+^) at a concentration of 1 mmol/L were added to 200 μL of a stock solution of Probe A, and no significant fluorescence enhancement occurred when other metal cations were added. However, after the addition of Al^3+^, significant fluorescence enhancement occurred at 450 nm. The ion selectivity of Probe B was investigated in the same way. As shown in Figure 3b, the fluorescence of the detection system was almost completely quenched at 413 nm when Fe^3+^ was detected, while no fluorescence quenching was observed when other ions were detected. The above results show that Probe A and Probe B can be used as selective fluorescence sensors for Al^3+^ and Fe^3+^, respectively.

As shown in Figure 3c, when the above solution mixture was stored under ultraviolet light (365 nm), the solution containing Al^3+^ exhibited bright blue fluorescence, while the fluorescence intensities of the other ions were not much different from those of the blank. Moreover, after measuring the 3D fluorescence spectra of Probe A aqueous solutions with and without 1 equiv of Al^3+^, Figure 3d,e show that the fluorescence intensity at the central position increased significantly after the addition of Al^3+^. As shown in Figure 3f, under irradiation with a portable UV black lamp (365 nm), the aqueous solution of Probe B emitted bright blue fluorescence, while the blue fluorescence disappeared after the addition of Fe^3+^, and the fluorescence intensity of other ions was not much different from that of the blank. The 3D fluorescence spectra of Probe B aqueous solutions with and without one equivalent Fe^3+^ were also recorded; as shown in Figure 3g,h, the fluorescence intensity in the central region was significantly weakened after the addition of Fe^3+^. Therefore, the above experiments show that Probe A and Probe B can selectively detect Al^3+^ and Fe^3+^ based on fluorescence colour changes.

### 2.3. Detection Limits of Fluorescent Probes for Al^3+^ and Fe^3+^

**Figure 4:**
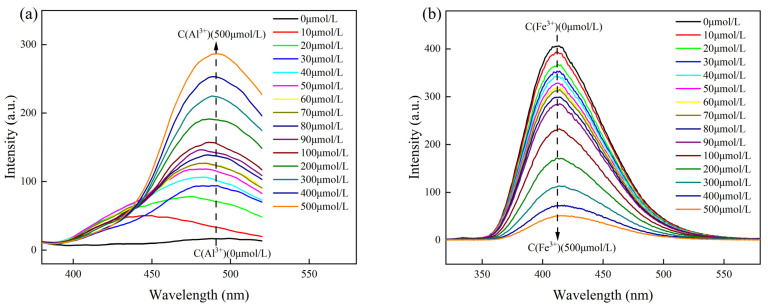

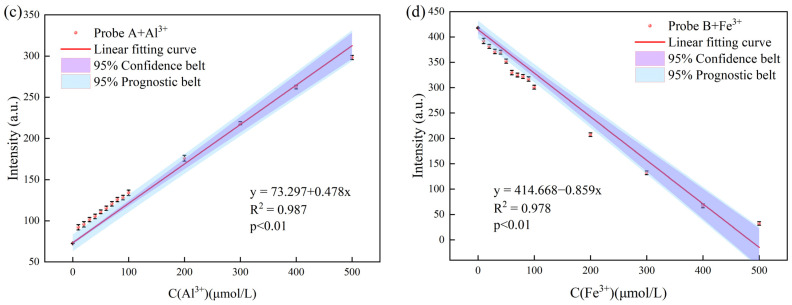
(**a**,**b**) Fluorescence spectra of Probe A and Probe B in the presence of different concentrations of Al^3+^ and Fe^3+^; linear relationships between (**c**) Al^3+^ concentration and Probe A fluorescence intensity and (**d**) Fe^3+^ concentration and Probe B fluorescence intensity.

The fluorescence responses of Probe A and Probe B stock solutions, each at a concentration of 200 μL, were studied by adding different concentrations of Al^3+^ and Fe^3+^ at an excitation wavelength of 300 nm to investigate their effects on the fluorescence spectrum. The results are shown in Figure 4.

As shown in Figure 4a, in the range of 20–500 μmol/L, because the linker group in the Probe A fluorescent molecule was a Schiff base, the molecule itself had an electron-rich structure [30]. Therefore, with the increasing Al^3+^ concentration in the detection system, the energy of the bonding electron pair of the fluorescent molecule changed, and the fluorescence intensity of Probe A increased. Probe B had an amide structure consisting of nitrogen and oxygen atoms, and the probe molecule itself had a higher-energy nonbonding electron pair [31]. Therefore, as shown in Figure 4b, the fluorescence intensity of Probe B exhibited a continuous decrease as the concentration of Fe^3+^ in the detection system increased.

In addition, working curves were plotted using the Al^3+^ and Fe^3+^ ion concentrations as the abscissa and the fluorescence values of the highest peak of the fluorescence spectrum of the detection system as the ordinate; then, the detection limits were determined by fitting. As shown in Figure 4c,d, the slopes of the linear curves were 0.478 and −0.859, with correlation coefficients of 0.987 and 0.978, respectively, showing a good linear relationship. Moreover, the standard deviation of the blank response of probe A and probe B was measured by six parallel experiments, and the standard deviation σ was calculated to be 0.188 and 0.482, respectively. According to the standard formula for the calculation of the detection limit [32]

LOD = 3*σ*/*S* (1)

the detection limits of Probe A and Probe B were calculated to be 1.180 and 1.683 μmol/L, respectively. We carried out variance analysis on the above experimental data. When probe A detected different concentrations of Al^3+^ and probe B detected different concentrations of Fe^3+^, the measured F values were 1034 and 571, respectively, and the corresponding *p* values were 8.888 × 10^−14^ and 3.971 × 10^−12^, respectively. The *p* values were all less than the significance level of 0.01, and the 95% confidence intervals (71.451, 75.144 and 407.450, 421.885) indicated that there were significant differences in the data within the group and the correlation was strong.

The above experimental results show that the detection method based on the 100% water-soluble Probe A and Probe B can be used for the detection of trace Al^3+^ and Fe^3+^ in high-purity lithium carbonate (where σ is the standard deviation of the results of multiple measurements of the blank response, and S is the slope of the calibration curve).

### 2.4. Stability and Response Time of Probe A and Probe B

**Figure 5:**
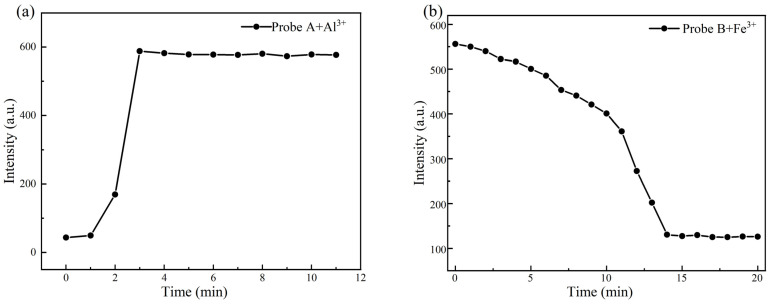
(**a**) Effect of reaction time on the fluorescence intensity of the Probe A+Al^3+^ system; (**b**) effect of reaction time on the fluorescence intensity of the Probe B+Fe^3+^ system.

To determine the selectivity and responsiveness of Probe A to Al^3+^ and Probe B to Fe^3+^, the changes in the fluorescence spectra over time were determined after adding the probe stock solutions to the ions to be measured, and the results are shown in Figure 5.

As shown in Figure 5a, when Probe A was used to detect Al^3+^, the fluorescence intensity of the system gradually increased with time, and reached a stable value within ~3 min. However, when Probe B was used to detect Fe^3+^ (Figure 5b), the fluorescence intensity of the system gradually decreased over time, reaching a stable value within ~14 min. Probe A and Probe B completed the specific detection of lithium carbonate aqueous solution containing Al^3+^ and Fe^3+^ within a time range of 10 and 30 s, respectively, indicating a very fast detection process of Al^3+^ and Fe^3+^. In summary, the detection method based on a water-soluble fluorescent probe can directly detect Al^3+^ and Fe^3+^ impurities in a lithium carbonate aqueous solution; compared with other detection methods, the identification process is faster, the detection steps are simpler, and the detection efficiency is further improved, indicating that this method can achieve the rapid detection of Al^3+^ and Fe^3+^ in high-purity lithium carbonate.

**Figure 6:**
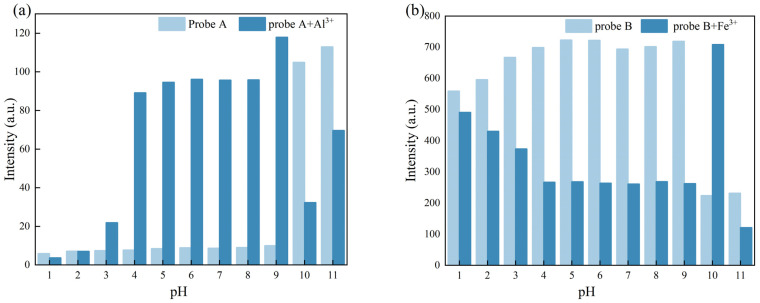
(**a**) Effect of pH on the fluorescence intensity of Probe A and Probe A+Al^3+^ systems; (**b**) effect of pH on the fluorescence intensity of Probe B and Probe B+Fe^3+^ systems.

Schiff base compounds tend to hydrolyse upon changing the pH value of the medium. However, many Schiff bases have been reported to be very stable over a wide pH range [33,34,35]. To assess their stability at varying pH, the fluorescence spectra of the probes and the corresponding complexes were recorded at different pH values, and the results are shown in Figure 6.

As shown in Figure 6a, the fluorescence intensity of Probe A remained stable in the pH range of 1.0–9.0. However, after the addition of Al^3+^, the fluorescence intensity gradually increased at pH values between 1 and 4 and remained unchanged in the pH range of 4.0–9.0. As shown in Figure 6b, the fluorescence intensity of Probe B increased gradually with increasing pH, and remained stable in the pH range of 4.0–9.0; however, upon Fe^3+^ addition, the fluorescence decreased with increasing pH and remained stable in the pH range of 4.0–9.0. Therefore, the above experiments show that the Probe A+Al^3+^ and Probe B+Fe^3+^ complexes remained stable in the 4.0–9.0 pH range.

### 2.5. Exploration of Fluorescence Reversibility of Probe A and Probe B

**Figure 7:**
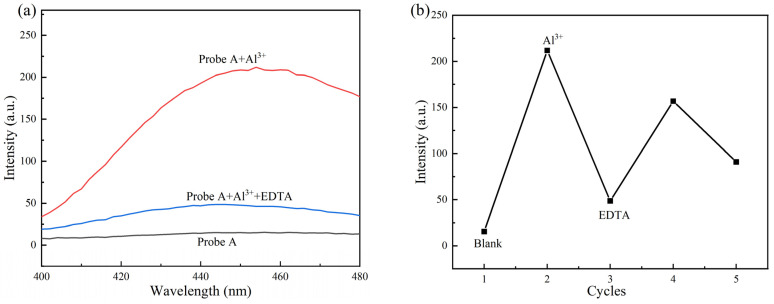
Fluorescence spectrum (**a**) and fluorescence intensity change (**b**) after successive additions of Al^3+^ and EDTA in Probe A solution.

**Figure 8:**
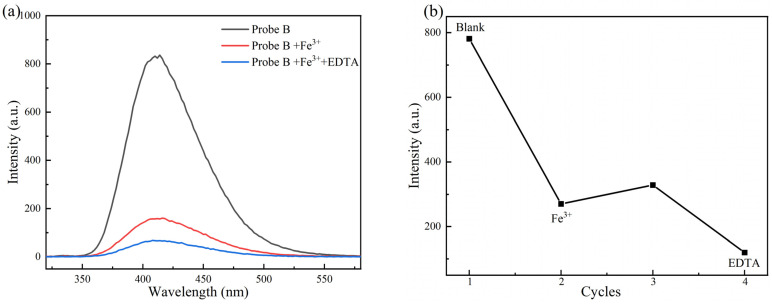
Fluorescence spectrum (**a**) and fluorescence intensity changes (**b**) of Fe^3+^ and EDTA added to Probe B solution.

To fully understand the responses of Probe A to Al^3+^ and Probe B to Fe^3+^, further experiments were conducted to explore the reversibility of the corresponding processes. Al^3+^ and EDTA were successively added to an aqueous solution of Probe A, whereas Fe^3+^ and EDTA were added to an aqueous solution of Probe B, followed by repeated measurements of the fluorescence intensity. The results are shown in Figure 7 and Figure 8, respectively.

As shown in Figure 7, no fluorescence was observed in the initial solution; the fluorescence intensity reached ~200 after the addition of Al^3+^, dropped to 40 after the addition of EDTA, and returned to ~170 when Al^3+^ was added again. These results show that the designed probe molecule can reversibly detect Al^3+^ ions through the coordination of EDTA and Al^3+^, which confirms that it can be reused.

As shown in Figure 8, the fluorescence value of the initial Probe B solution is as high as 800, which decreases to 150 with the addition of Fe^3+^, and then the fluorescence intensity decreases even more after EDTA is added. These results showed that the coordination effect between the designed Probe B molecule and Fe^3+^ was stronger than that of EDTA and Fe^3+^. The Probe B molecule is not reversible in the detection of Fe^3+^, and it is not possible to reuse probe molecules.

### 2.6. Determination of Binding Mode of Probes and Ions

**Figure 9:**
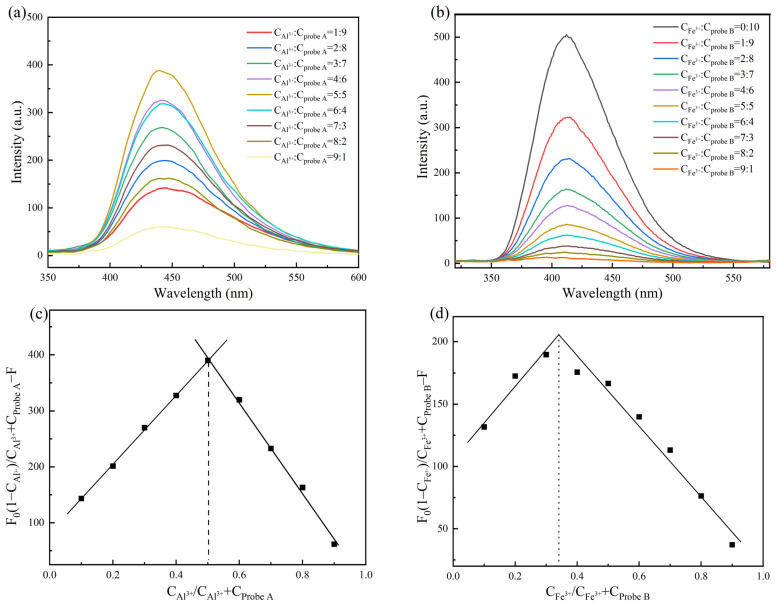
(**a**) Fluorescence spectra of Probe A and Al^3+^ in different ratios in H_2_O solution; (**b**) job diagram of Probe A combined with Al^3+^; (**c**) fluorescence spectra of Probe B and Fe^3+^ mixed in different ratios in H_2_O solution; (**d**) Job plot of Probe B binding to Fe^3+^.

The combined stoichiometry of Probe A+Al^3+^ and Probe B+Fe^3+^ was determined using the Job’s plot method. The total concentration of the solution was kept at 10^−3^ mol/L, the probe to ion ratio in the mixed solution was changed, the fluorescence spectrum of each group of samples was measured, and the fluorescence intensities of Probe A+Al^3+^ and Probe B+Fe^3+^ were recorded at 413 and 450 nm, respectively. Job curves were plotted for the two complexes according to different ratios and fluorescence intensities, and the experimental results are shown in Figure 9.

As can be seen from Figure 9a, the fluorescence intensity of the mixture is maximum when the ratio of Probe A+Al^3+^ reaches 1:1, and the maximum value is shown when the molar fraction of Probe A detects Al^3+^ at 0.5 as shown in Figure 9c. The binding stoichiometry of Probe B and Fe^3+^ was studied in the same way; as shown in Figure 9b,d, the fluorescence intensity of the mixture decreased with the decreasing proportion of Probe B+Fe^3+^ in the mixed system, and a molar fraction of 0.33 was observed when Probe B detected Fe^3+^. These results show that the binding stoichiometric ratios of Probe A+Al^3+^ and Probe B+Fe^3+^ in aqueous solution were 1:1 and 2:1, respectively.

**Figure 10:**
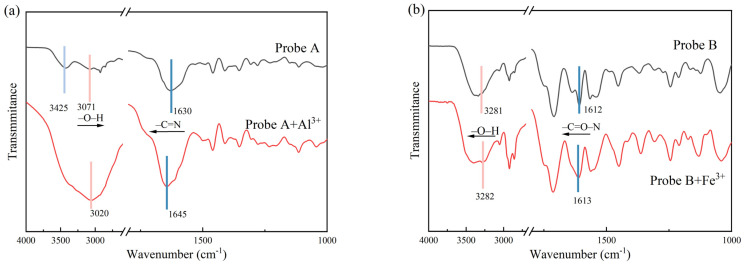
(**a**) Infrared spectra of Probe A and the Probe A+Al^3+^ complex; (**b**) infrared spectra of Probe B and the Probe B+Fe^3+^ complex.

To further explore the binding mode of the probe to the ions, the binding sites of Probe A+Al^3+^ and Probe B+Fe^3+^ were investigated by infrared spectroscopy. As shown in Figure 10a, the infrared spectrum of Probe A exhibited significant changes at 1630, 3071, and 3425 cm^−1^ when Al^3+^ was added to its solution. Among these changes, the characteristic peak of the Schiff base (–CN=N) at 1630 cm^−1^ shifted to 1645 cm^−1^; the shift to higher wavenumbers indicates that the nitrogen atom on the Schiff base participated in the coordination. The –O–H peaks at 3425 and 3071 cm^−1^ moved to 3020 cm^−1^; the shift to lower wavenumbers indicates that the electronegative oxygen atoms on the hydroxyl group were involved in the coordination.

As shown in Figure 10b, the infrared signals at 1612 and 3281 cm^−1^ significantly changed when Fe^3+^ was added to Probe B. Among them, the characteristic peak of the amide bond (–C=O–N) shifted from 1612 to 1613 cm^−1^; the shift to higher wavenumbers indicates that the nitrogen atom on the amide bond (–C=O–N) was involved in the coordination of Fe^3+^. The –O–H vibration peak at 3281 cm^−1^ also shifted to a higher wavenumber (3282 cm^−1^), showing that the oxygen atom of –O–H was involved in the coordination. In summary, it can be inferred that the binding sites of Probe A to Al^3+^ were the nitrogen atom of the Schiff base, and the oxygen atoms of the phenolic and carboxyl hydroxyl groups, whereas the binding sites of Probe B to Fe^3+^ were the O atom of the amide bond (–C=O–N), the N atom, and the oxygen atom of –O–H [36,37,38,39].

### 2.7. Simulation Analysis of Response Mechanisms

**Figure 11:**
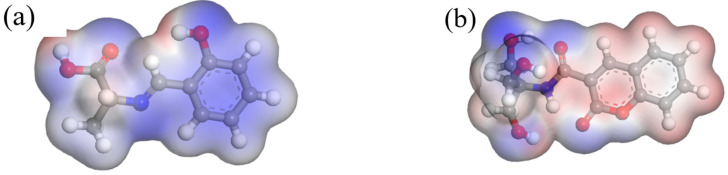
(**a**) Electrostatic potential of Probe A; (**b**) electrostatic potential of Probe B.(In the molecular structure formula, red, white, gray and blue correspond to oxygen, hydrogen, carbon and nitrogen, respectively.)

**Figure 12:**
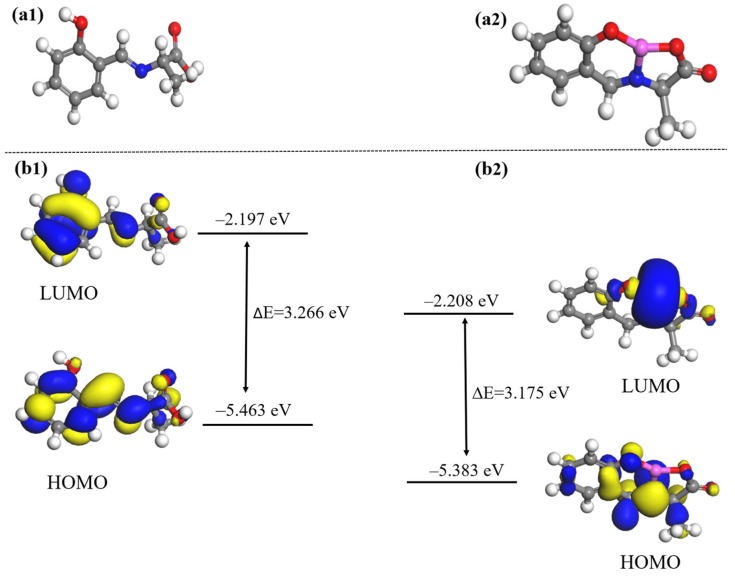
(**a1**,**a2**) Optimised structures of Probe A and Probe A+Al^3+^; (**b1**,**b2**) FMOs of Probe A and Probe A+Al^3+^ in different electronic states.

**Figure 13:**
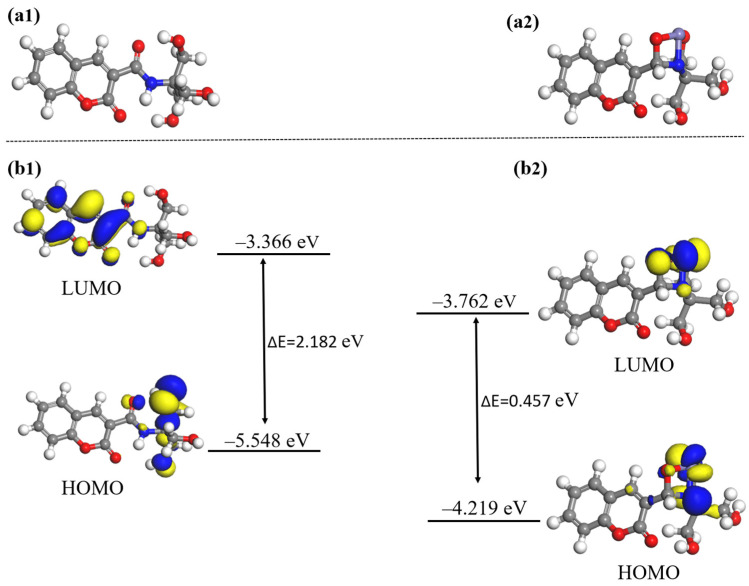
(**a1**,**a2**) Optimised structures of Probe B and Probe B+Fe^3+^; (**b1**,**b2**) FMOs of Probe B and Probe B+Fe^3+^ in different electronic states.

In this paper, in order to further determine the reaction sites between Probe A and Al^3+^, and Probe B and Fe^3+^, water was used as the solvent, and the DFT method was combined with the Perdew–Burke–Ernzerhof (PBE) functional description under the generalized gradient approximation (GGA). The double numerical plus polarization (DNP) basis set was used to theoretically study the fluorescent probe and its complex molecules [40,41,42,43]. The experimental structure is shown in Figure 11, Figure 12 and Figure 13.

The electrostatic potential surface of the molecule is marked with different colours, with blue and red representing the negative and positive potential regions, respectively. As seen from the structures of Figure 11a,b, both Probe A and Probe B have very tensile structures, in which N, –OH, and –C=O–N are blue negative potential regions. In general, the ESP value in the negative potential region is small, and the corresponding atoms are also considered to have a greater probability of participating in the electrophilic reaction. In this study, the Al^3+^ and Fe^3+^ metal ions to be detected were positively charged, making it easier to deprotonate and coordinate the negatively charged nitrogen atoms and phenolic hydroxyl groups in the probe molecule [44,45]. Therefore, it is likely that Probe A is linked to Al^3+^ on the nitrogen atom on the Schiff base, and the oxygen atom on the phenolic hydroxyl group, and the oxygen atom on the O, N, and –O–H groups on the –C=O–N on Probe B is linked to Fe^3+^.

In order to further evaluate the response mechanism of Probe A+Al^3+^ and the origin of the enhancement of the fluorescence emission, the fluorescence mechanism of Probe A+Al^3+^ for Al^3+^ and Fe^3+^ was elucidated through density functional theory calculations. First, we investigated the optimised structures of Probe A and Probe A+Al^3+^ (Figure 12a); according to the optimised configuration calculated, shown as ball-and-stick models in Figure 12(a1,a2), Al^3+^ formed intramolecular hydrogen bonds after combining with Probe A. Next, we analysed the geometry-optimised structures and energies of the highest occupied molecular orbital (HOMO) and lowest unoccupied molecular orbital (LUMO) of Probe A and Probe A+Al^3+^ (Figure 12b). Probe A and Probe A+Al^3+^ had HOMO–LUMO energy gaps of 3.266 and 3.175 eV, respectively. In Probe A, the LUMO was distributed on the phenol moiety, while the HOMO extended over the entire probe molecule. After combining with Al^3+^, the LUMO orbital was centred on Al^3+^ and distributed on the amide moiety, while the HOMO orbital was mainly located in the salicylaldehyde moiety; moreover, the phenolic hydroxyl group captured the hydrogen atom of the carboxyl hydroxyl group, which led to the combination of aluminium ions with the oxygen atoms of the phenolic hydroxyl and carboxyl hydroxyl groups of Probe A [46,47,48].

Figure 13 shows the optimised structures of Probe B before and after the addition of Fe^3+^. As shown in Figure 13(a1,a2), the molecular configuration of Probe B changed significantly after the addition of Fe^3+^, due to the isomerisation and rotation of the C–N bond. In addition, the HOMO and LUMO energy levels, which characterise the electron donor and acceptor signatures of the molecule, were calculated for Probe B and Probe B+Fe^3+^. As shown in Figure 13b, the energies of Probe B and Probe B+Fe^3+^ in the excited state were 2.182 and 0.457 eV, respectively; moreover, the absorption peak of Probe B+Fe^3+^ was red-shifted compared with that of Probe B, and the HOMO–LUMO energy gap difference between Probe B and Probe B+Fe^3+^ was reduced. This indicates a lower stability of the probe+Fe^3+^ complex, corresponding to a higher reactivity, which promotes the photoelectron transfer (PET) process.

The above theoretical results show that, compared with Probe A, the HOMO–LUMO energy gap of Probe A+Al^3+^ decreases by 0.091 eV after the addition of Al^3+^, indicating that the energy of Al^3+^ decreased after binding to the probe, reaching a steady state. The enhanced fluorescence emission of Al^3+^ can be attributed to the excited intramolecular proton transfer (ESIPT) effect [49]. The addition of Fe^3+^ to the aqueous solution of Probe B resulted in the cleavage of the C–N bond in the probe molecule and the accumulation of electrons on the amino and ketone groups, which were responsible for turning off the fluorescence upon binding via the PET mechanism [50,51]. As a result, Probe B exhibited fluorescence quenching when detecting Fe^3+^.

### 2.8. Practical Detection of Impurity Ions in High-Purity Lithium Carbonate

**Figure 14:**
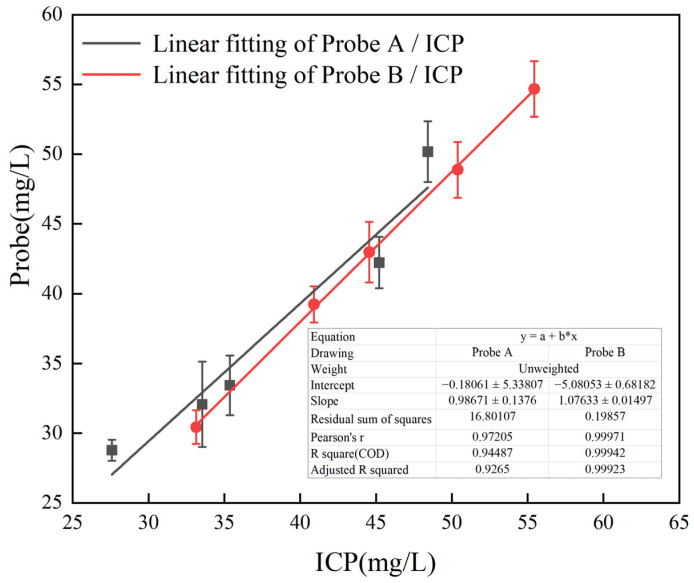
Results of Al^3+^ and Fe^3+^ content determination in the sample using two different methods.

To evaluate the practical application value of Probe A and Probe B for metal ion detection, five groups of samples were collected at different times from the lithium carbonate production line in the pilot plant located at Qinghai Salt Lake. The samples diluted 10 times were separately added to Probe A and Probe B; then, the fluorescence of Probe A+Al^3+^ and Probe B+Fe^3+^ was measured and confirmed by ICP-OES. The results are shown in Table 1 and Table 2 and Figure 14.

The results of Al^3+^ detection by probe A are shown in Table 1, and Figure 14 shows the results obtained by fitting the results of probe A and ICP. The correlation coefficient R^2^ between the fluorescence probe and the ICP-OES analysis method was 0.945. The results of Fe^3+^ detected by probe B are shown in Table 2, and the results of the two detection methods are shown in Figure 14. The R^2^ correlation coefficient of the two analysis methods was 0.999. The above studies have confirmed that the two methods have good consistency.

In order to further explore the applicability of the probe to the detection of impurity ions in high-purity lithium carbonate, we conducted an independent sample t-test analysis of the above experimental data. Table 3 and Table 4 are the group statistical results and independent sample t-test results of probe A and ICP-OES in the detection of Al^3+^. The mean ± standard deviation of Al^3+^ detected by probe A and ICP-OES were 38.02 ± 8.60 and 37.34 ± 8.73, respectively. From the significance *p* = 0.98 > 0.05 in the Levin variance equivalence test in Table 3, it can be seen that the variance of the test results of the two detection methods is equal. Taking the test results of the first row of Table 3 as the standard, *p* = 0.90 > 0.05 means that there is no significant difference between the average value of Al^3+^ detected by probe A and the average value of ICP-OES detection results. At the same time, the 95% confidence interval of the difference under the t-test is (−11.95, 13.32), indicating that 95% of the confidence believes that the true difference between the overall mean values measured by the two methods is between −11.95 and 13.32, and the interval contains 0; therefore, it is once again proved that there is no difference between the measured values of the two detection methods.

Table 4 shows the independent sample t-test results of probe B and ICP-OES in detecting Fe^3+^. The mean ± standard deviation of Fe^3+^ detected by probe B and ICP-OES were 44.89 ± 8.59 and 43.24 ± 9.25, respectively. The significance of *p* = 0.90 > 0.05 in the variance equivalence test shows that the variance of the two detection methods is equal. Taking the test results of the first row of Table 4 as the standard, *p* = 0.78 > 0.05 means that there is no significant difference between the average value of Fe^3+^ detected by probe B and the average value of Fe^3+^ detected by ICP-OES. At the same time, it can be seen from Table 4 that the 95% confidence interval of the difference under the t-test is (−11.37, 14.67). There is 95% confidence that the true difference between the overall mean values measured by the two methods is between −11.37 and 14.67, and the interval contains 0, therefore, it is once again proved that there is no difference between the measured values of the two detection methods.

These results show that the detection results of probe A and probe B are not significantly different from those of ICP-OES, so probe A and probe B are suitable for the ultrasensitive detection of Al^3+^ and Fe^3+^ in complex samples.

## 3. Materials and Methods

### 3.1. Reagents

The following reagents were used: coumarin-3-carboxylic acid (97%), 3-hydroxyaminomethane (99%), dichloromethane (99%), deuterated dimethyl sulfoxide (99%), deuterated chloroform (99%), methanol (analytically pure), ethanol (98%), salicylaldehyde (99%), dl-alanine (99%),The above reagents were purchased from Shanghai McLin Biochemical Technology Co., Ltd., Shanghai, China. Magnesium chloride hexahydrate (analytically pure), anhydrous ferric chloride (analytically pure), lithium chloride monohydrate (analytically pure), and aluminium chloride hexahydrate (analytically pure),the above reagents were purchased from Sinopharm Chemical Reagent Co., Ltd., Shanghai, China.

### 3.2. Analysis and Characterisation Methods

A UV/Vis spectrophotometer (UV-26001, Agilent Shanghai Co., Ltd., Shanghai, China), a fourier transform infrared absorption spectrometer (NVENIO-S, Brooke, Germany), a fluorescence spectrometer (Cary Eclipse, Agilent, Santa Clara, CA, USA),an inductively Coupled Plasma Optical Emission Spectrometer (ICP-OES) (5800VD, Agilent, Santa Clara, CA, USA) were used for analysis.

## 4. Conclusions

In the production process of high-purity lithium carbonate, the contamination of alloy metal ions such as iron and aluminium in the lithium salt is one of the issues affecting the quality of the lithium carbonate products, which limits their subsequent application. However, methods for the rapid detection of metal ions in aqueous environments have been lacking for many years. To solve this problem, this work presents a method for the rapid detection of trace iron and aluminium impurities in high-purity lithium carbonate using water-soluble fluorescent probes.

First, a method involving the grafting of hydrophilic groups was employed to prepare the 100% water-soluble Probe A and Probe B. We found that Probe A and Probe B had specific responses to Al^3+^ and Fe^3+^, respectively, as well as high sensitivity and selectivity for these ions in the 4–9 pH range in aqueous solution, along with detection limits of 1.180 and 1.683 μmol/L and response times as low as 10 and 30 s, respectively. Then, the stoichiometric ratios of Probe A+Al^3+^ and Probe B–Fe^3+^ were calculated by Job’s plot method to be 1:1 and 2:1, respectively. The coordination structures of Probe A-Al^3+^ and Probe B+Fe^3+^ were characterised by FT-IR and ESP analyses, and optimised via calculations. The experimental results showed the occurrence of the ESIPT effect when Probe A detected Al^3+^; moreover, the electrons were responsible for turning off the fluorescence when Probe B detected Fe^3+^ through the PET mechanism. At the same time, the concentrations of Al^3+^ and Fe^3+^ in practical samples were quantitatively determined, and the results of the fluorescent probe method were validated by ICP-OES. The analysis showed that the linear relationship slopes of probe A and probe B with ICP were 0.945 and 0.999, respectively, confirming that the fluorescent probe and ICP-OES methods yielded consistent results. In summary, the results show that the present method can be used for the rapid detection of trace iron and aluminium impurities in high-purity lithium carbonate.

## Figures and Tables

**Table 1:** Results of two methods for determining the Al^3+^ content in samples.

	ICP (mg/L)	Probe A Fluorescence Method (mg/L)
Sample 1	35.36	33.44
Sample 2	27.59	28.78
Sample 3	48.42	50.17
Sample 4	33.54	32.07
Sample 5	45.21	42.23
Mean value	38.02	37.34
Standard deviation	8.60	8.73
Average standard error	3.85	3.90

**Table 2:** Results of two methods for determining Fe^3+^ content in samples.

	ICP (mg/L)	Probe B Fluorescence Method (mg/L)
Sample 1	33.15	30.44
Sample 2	50.39	48.87
Sample 3	55.44	54.67
Sample 4	44.56	42.97
Sample 5	40.91	39.23
Mean value	44.89	43.24
Standard deviation	8.59	9.25
Average standard error	3.84	4.14

**Table 3:** Al^3+^ independent sample test.

		Levin Variance Equivalence Test		Mean Equivalence Test						
		F	Significance	*t*	Degree of Freedom	Significance (Two-Tailed)	Mean Value Difference	Standard Error Difference	Difference 95% Confidence Interval	
									Lower Limit	Upper Limit
mg/L	Assume equal variance	0.001	0.98	0.13	8	0.90	0.69	5.5	−11.95	13.32
	No assumption of equal variance			0.13	8	0.90	0.69	5.5	−11.95	13.32

**Table 4:** Fe^3+^ independent sample test.

		Levin Variance Equivalence Test		Mean Equivalence Test						
		F	Significance	t	Degree of Freedom	Significance (Two-Tailed)	Mean Value Difference	Standard Error Difference	Difference 95% Confidence Interval	
									Lower Limit	Upper Limit
mg/L	Assume equal variance	0.017	0.90	0.29	8.0	0.78	1.65	5.65	−11.37	14.67
	No assumption of equal variance			0.29	8.0	0.78	1.65	5.65	−11.38	14.69

## Data Availability

The data can be obtained from the author.
